# Identification and functional deciphering suggested the regulatory roles of long intergenic ncRNAs (lincRNAs) in increasing grafting pepper resistance to *Phytophthora capsici*

**DOI:** 10.1186/s12864-021-08183-z

**Published:** 2021-12-02

**Authors:** Junliang Yin, Jiahui Yan, Lu Hou, Liling Jiang, Wenrong Xian, Qingyun Guo

**Affiliations:** 1 Qinghai Academy of Agriculture and Forestry Science, Key Laboratory of Agricultural Integrated Pest Management, State Key Laboratory of Plateau Ecology and Agriculture, Qinghai University, Qinghai University, 810016 Xining, Qinghai Province China; 2 Hubei Key Laboratory of Waterlogging Disaster and Agricultural Use of Wetland, College of Agriculture, Yangtze University, 434000 Jingzhou, Hubei China; 3 Qinghai Academy of Agriculture and Forestry Science, Qinghai University, 810016 Xining, China

**Keywords:** ncRNAs, *Capsicum annuum*, *cis*-regulating, lincRNA-miRNA-mRNA network

## Abstract

**Background:**

As a popular and valuable technique, grafting is widely used to protect against soil-borne diseases and nematodes in vegetable production. Growing evidences have revealed that long intergenic ncRNAs (lincRNAs) are strictly regulated and play essential roles in plants development and stress responses. Nevertheless, genome-wide identification and function deciphering of pepper lincRNAs, especially for their roles in improving grafting pepper resistance to *Phytophthora capsici* is largely unknown.

**Results:**

In this study, RNA-seq data of grafting and control pepper plants with or without *P*. *capsici* inoculation were used to identify lincRNAs. In total, 2,388 reliable lincRNAs were identified. They were relatively longer and contained few exons than protein-coding genes. Similar to coding genes, lincRNAs had higher densities in euchromatin regions; and longer chromosome transcribed more lincRNAs. Expression pattern profiling suggested that lincRNAs commonly had lower expression than mRNAs. Totally, 607 differentially expressed lincRNAs (DE-lincRANs) were identified, of which 172 were found between *P*. *capsici* resistance grafting pepper sample GR and susceptible sample LDS. The neighboring genes of DE-lincRNAs and miRNAs competitively sponged by DE-lincRNAs were identified. Subsequently, the expression level of DE-lincRNAs was further confirmed by qRT-PCR and regulation patterns between DE-lincRNAs and neighboring mRNAs were also validated. Function annotation revealed that DE-lincRNAs increased the resistance of grafting prepper to *P*. *capsici* by modulating the expression of disease-defense related genes through *cis*-regulating and/or lincRNA-miRNA-mRNA interaction networks.

**Conclusions:**

This study identified pepper lincRNAs and suggested their potential roles in increasing the resistance level of grafting pepper to *P*. *capsici*.

**Supplementary Information:**

The online version contains supplementary material available at 10.1186/s12864-021-08183-z.

## Background

In recent years, a growing body of studies has demonstrated that eukaryotic genomes contain many regions that transcribe functional noncoding RNAs (ncRNAs), which are typically grouped into housekeeping and regulatory ncRNAs [1]. Among the regulatory ncRNAs, long ncRNAs (lncRNAs) have been suggested to be the critical player during eukaryotic gene regulation [2]. According to the genomic transcription region and the transcript length, plant long ncRNAs can be classified into four types, including long intergenic ncRNAs (lincRNAs), long intron ncRNAs, natural antisense long noncoding RNAs (lncNATs), and promoter lncRNAs [2]. The identification, characterization and deciphering of the possible biological functions for lncRNAs has been a rapidly developing research area over the past decade [3]. An increasing number of lincRNAs have been showed to play essential regulatory roles in higher eukaryotic organisms [4]. Until now, the identification and characterization of lincRNAs have been performed in humans, zebrafish, fruit flies and chickens using high-throughput RNA-seq [2]. Compared to human and animal studies, studies focusing on plant lincRNAs started relatively late. Nevertheless, recent studies have reported many ncRNAs in plants, such as potato lincRNAs responsive to *Pectobacterium carotovorum* subspecies *brasiliense* infection [5], wheat lincRNAs induced by stripe rust and powdery mildew disease [2], *Populus* lincRNAs involving in drought-responsive [6], maize lincRNAs regulating growth and development [7], and *Arabidopsis* lincRNAs participating in low-nutrient response [8]. However, few studies focusing on genome-wide identification and characterization of pepper lincRNAs have been performed using large-scale RNA-seq data.

Peppers (*Capsicum* spp., mainly *C. annuum* L.) are economically and socially important vegetables that are widely cultivated in most countries all over the world [9]. In China, they are grown on a total of 2.1 million ha, and the annual output value is over 40 billion US dollars [10]. However, the safety and sustainable production of pepper was seriously threatened by late blight disease caused by the soil-borne pathogen *Phytophthora capsici* [11]. Worldwide annual losses due to this disease are more than 100 million US dollars [12]. Currently, the management of *P*. *capsici* largely relies on chemical and cultural practices, including fungicide applications, soil solarization, and crop rotation, as well as irrigation management [12]. Grafting is one of the most popular and valuable techniques used to protect against soil borne-diseases and nematodes in the vegetable production [13]. Compared to numerous years of breeding and biotechnological programs, this technique is simple, convenient and environmental-friendly, and grafting susceptible commercial cultivars onto resistant rootstocks can reduce the negative effects of biotic and abiotic stress [14]. Therefore, the grafting technique is widely used all over the world on a commercial scale in several economically important vegetables, such as tomato, cucumber, and watermelon [9].

Grafting pepper to resistant or partially resistant rootstocks has resulted in disease reduction [14]. For example, Jang, et al. [15] found grafting pepper showed greater resistance to both *Phytophthora* blight and bacterial wilt without any negative effect on yield and fruit quality. Oka, et al. [16] reported that proper combination of rootstocks and scions could constrain nematode infection and proliferation and increase pepper yield. Nevertheless, studies focusing on physiological and agronomical responses in grafted pepper plants are still rarely performed, and their physiology and biochemistry responses when subjected to biotic and abiotic stress have also been insufficiently investigated. Therefore, it is necessary to explore the underlying mechanisms by which grafting alleviates stress, as it is crucial for carrying out more phenotypical screenings of various rootstock-scion combinations. Previously, we found that using cv. ‘Jingxin No. 5’ as rootstocks can significantly improve the resistance level of cv ‘Ledu’ scion to *P*. *capsici*, the causal agent of pepper late blight [17]. However, the physiological and molecular mechanisms involved in scion and pepper rootstock interaction under *Phytophthora* blight stress are still unknown. Previous studies suggested that lincRNAs participated in the responding of pepper to abiotic stress conditions such as heat, cold, osmotic, and salinity [18], as well as in regulating fruit development [19] and ripening [20]. However, the roles of pepper lincRNAs in mediating disease resistance, especially for their roles in improving grafting pepper resistance to *Phytophthora capsici*, is largely unknown currently. Thus, in this study, genome-wide identification and characterization of pepper lincRNAs were performed. After confirming the reliability of RNA-seq data by qRT-PCR, attention was given to the expression pattern profiling and functional deciphering of lincRNAs, especially for lincRNAs that are responsible for increasing pepper resistance to *P*. *capsici* after grafting. Our results revealed the characterization of pepper lincRNAs and suggested their potential regulatory role in increasing the resistance of grafted pepper to *P. capsici*.

## Materials and methods

### Plant material and pathogen inoculation

Healthy and uniform pepper seeds (susceptible *Capsicum annuum* L. cv. ‘Ledu’ was used as scion and resistant cv. ‘Jingxin No. 5’ was used as rootstock) supplied by Qinghai Academy of Agriculture and Forestry Sciences were pre-germinated in an incubator and planted in a solar greenhouse. Four to six leaves seedlings with stem diameters larger than 2 mm were chosen for grafting. Split-grafting was applied to construct hetero-grafted pepper seedlings. Briefly, the upper parts of rootstocks were cut off, with the first pair of true leaves remaining, and the stem was separated from the middle with a 1 cm notch. The lower part of the scion was cut off, and three to four true leaves were maintained. Subsequently, the stem of the scion was cut into a 1 cm wedge and inserted into the notch of the rootstock. About 20 days later, grafting and nature growing pepper plants were used for inoculation and sampling.

For pathogen inoculation, *P. capsici* isolate PcXN1314, which was isolated from disease leaves collected in Xining city (E 101.7434, N 36.6722) in 2013 and showed high aggressiveness to cv. ‘Ledu’, was cultured on RSA (rye sucrose agar) medium in a dark climate chamber at 22°C for two weeks. Then plates were washed with cold sterile distilled water to harvest sporangia. The sporangia were counted using hemocytometer under a microscope and suspension was adjusted to 15,000 spores per mL, and incubated at 4℃ for 2 h to release motile zoospores. Then zoospores suspension was sprayed on pepper leaves. Three days after inoculation, three kinds of leaf samples, *P*. *capsici* inoculation ‘Ledu’ scion with ‘Jingxin No. 5’ rootstocks (GR), *P*. *capsici* inoculation ‘Ledu’ (LDS), and control of non-inoculated ‘Ledu’ (CK), were collected for RNA-seq sequencing. Each sample contains three biological replications.

### The lincRNA identification and function annnotation

RNeasy Mini Kit (QIAGEN, Beijing, China) was used to extract total RNA and RNase-free DNase I (Takara, Dalian, China) was used to degrade residual genomic DNA. After quality and quantity validation by Agilent 2100 Bioanalyzer (Agilent Technologies, Santa Clara, CA, USA) and Nanodrop 2000c Spectrophotometer (Thermo Fisher Scientific, Waltham, MA, USA), Ribo-Zero rRNA Removal Kit (Epicentre, Madison, WI, USA) was used to remove rRNA from total RNA, and then 1 µg of rRNA-depleted total RNA was used to prepare the sequencing library. Strand-specific 150 bp pair-end reads were produced on the Illumina HiSeq 2500 platform. Reads were deposited to the NCBI SRA database under project accession PRJNA728756 (https://www.ncbi.nlm.nih.gov/bioproject/?term=prjna728756). The sequenced raw reads were pre-processed to remove barcode and adaptor sequences and to remove low-quality and short reads [21]. Bowtie was used to filter out reads belonging to rRNAs. TopHat2 was used to map clean reads to the pepper reference genome *Capsicum*.*annuum*.L_Zunla-1_Release_2.0 [22]. Cufflinks was used to assemble transcripts and calculate the expression levels represented by FPKM values (fragments per kilobase of exon per million fragments mapped) [23]. Subsequently, the transcripts marked by Cufflinks classcode ‘u’ were kept and only transcripts with at least two reads were considered. After excluding the transcripts shorter than 200 bp, the remaining were further checked by CPC, CNCI, and Pfam to exclude potential protein-coding sequences [24]. The R package ‘gmodels’ [25] was used to perform principal component analysis (PCA), and ‘edgeR’ [26] was used to identify significantly differentially expressed genes and lincRNAs (filtering parameters FDR < 0.05 and |log2FC| > 1). Genes located in the 10 kb upstream or downstream of lincRNAs were manually extracted and functionally annotated by Blast2GO [27]. Several commonly used databases, namely Nr, Pfam, KOG/COG, and Swiss-Prot, were also used to perform gene function annotation [28]. To construct the possible circRNA-miRNA-mRNA regulation network, mature sequences of pepper miRNAs were collected from a previous study of Hwang, et al. [29]. Then, the lincRNA and miRNA sequences were analyzed by TargetFinder using default parameters to identify the identical alignment regions [30]. Meanwhile, pepper coding genes (mRNAs) and miRNAs sequences were analyzed by an online tool (http://plantgrn.noble.org/psRNATarget/) to identify the mRNAs targeted by the collected pepper miRNAs. The circRNA-miRNA-mRNA regulating network was illustrated by R package ‘ggalluvial’ [31].

### Expression pattern validation by quantitative real-time PCR (qRT-PCR)

The GR, LDS, and CK leaf samples collected for RNA-seq sequencing were also used to perform qRT-PCR analysis. Each sample contained three biological replicates. The synthesis of first-strand cDNA was conducted using the Prime-Script™ II First Strand cDNA synthesis kit (Takara Bio, Dalian, China) following manufacturer’s instruction [32]. The Primer Premier 5.0 (PREMIER Biosoft International, Palo Alto, CA, USA) was employed to design the primers, and their sequences are listed in Table S1. An ABI 7500 Real-Time PCR System (Thermo Fisher Scientific, Inc., Waltham, MA, USA) was used to perform qRT-PCR with the SYBR Premix Ex Taq™ II kit (Takara Bio, Dalian, China) [33]. Relative expression levels were calculated using the 2^−ΔΔCt^ method, and actin was used as the reference gene to normalize the expression level [34].

## Results and discussion

### Identification of lincRNAs from the pepper transcriptome

**Fig. 1:**
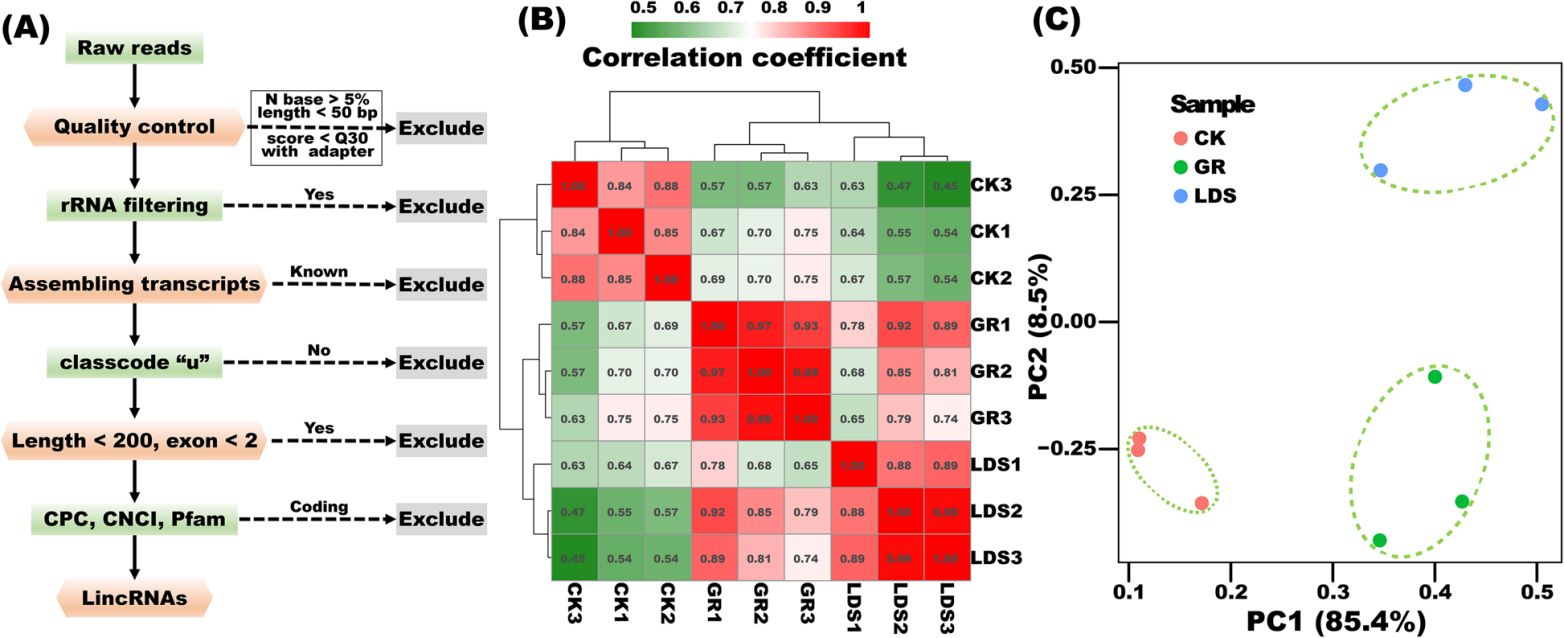
The workflow for lincRNA identification (**A**), and correlation analysis (**B**) and PCA (Principal Component Analysis) analysis (**C**) for validating the reliability of RNA-seq data. GR, LDS, and CK represent *P*. *capsici* inoculation ‘Ledu’ scion with ‘Jingxin No. 5’ rootstocks, *P*. *capsici* inoculation ‘Ledu’, and control of non-inoculated ‘Ledu’

RNA-seq has become a powerful tool for the identification of lincRNA [24]. To detect lincRNAs increasing pepper resistance to *P*. *capsici* after grafting, paired-end RNA-seq reads from CK, LDS, and GR were obtained. As shown in Fig. 1A, after quality control, 886 million 150 bp pair-end clean reads (99.90%) were kept. After mapping to rRNA, 76,644 reads were removed, and the reads left were mapped to the reference genome by TopHat2 and assembled by Cufflinks with default settings. In total, 52,872 transcripts were assembled and 22,072 were known transcripts [22]; the remaining 30,179 were new transcripts with Cufflinks classcodes ‘uijxceo’. Among them, 7,059 transcripts marked by classcode ‘u’ were retained. After filtering transcripts shorter than 200 bp and transcripts containing less than 2 exons, transcripts were further checked by CPC, CNCI, and Pfam to exclude potential protein-coding sequences. Finally, 2,388 reliable lincRNAs were identified (Table S2). Correlation and PCA analysis indicated a stable and distinct response of pepper plants to the corresponding treatment, as samples belonging to three treatments were separately distributed, and three biological replications belonging to the same treatment were clustered together (Fig. 1B, C).

### Characterization of pepper lincRNAs

**Fig. 2:**
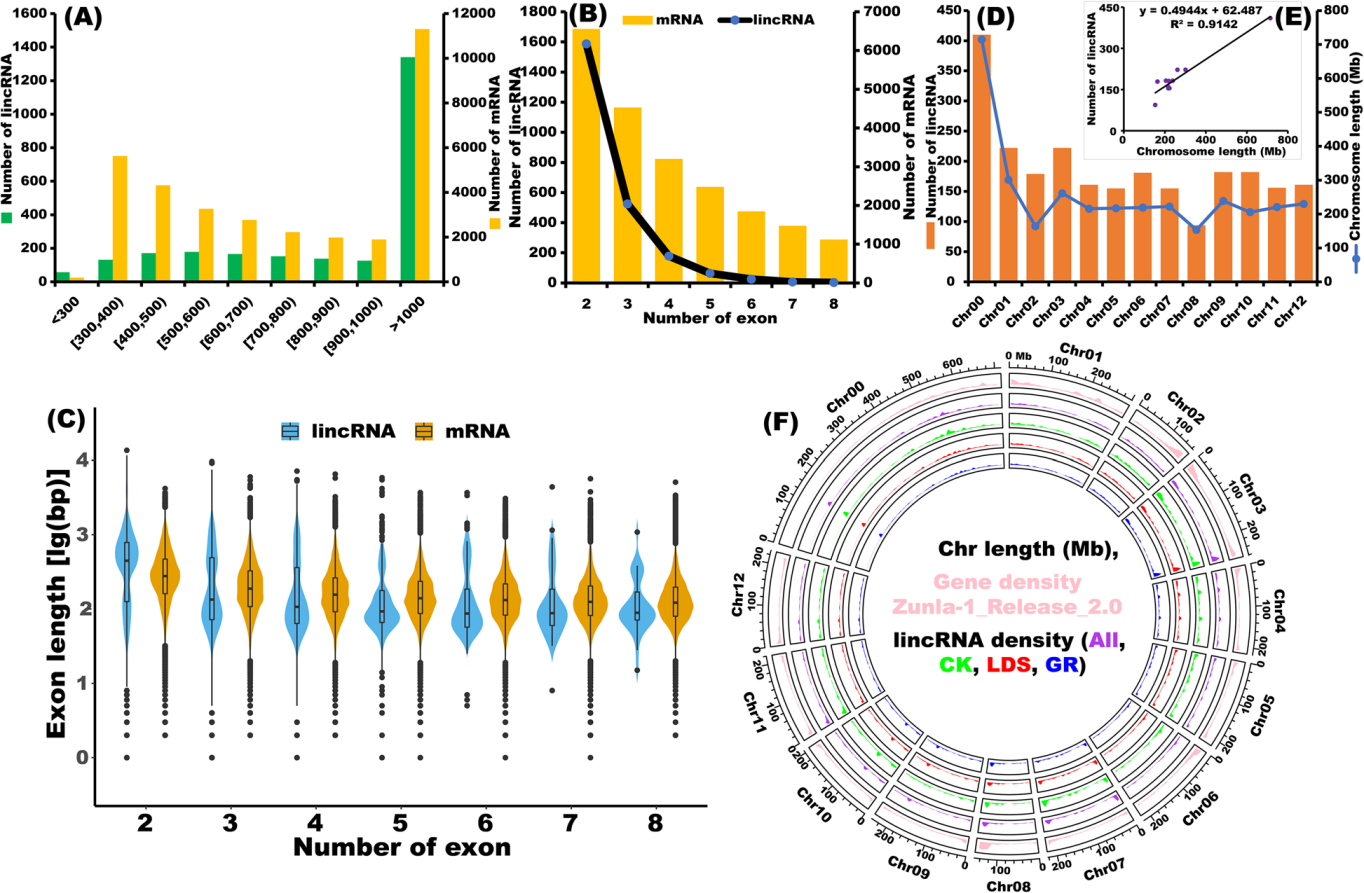
Characteristics of pepper lincRNAs. (**A**) Length distribution. Green and yellow histograms represent the member of lincRNA and mRNA belonging to the corresponding sequence length range. (**B**) Bias of exon number in lincRNAs. Black line and yellow histogram represent the member of lincRNA and mRNA containing corresponding number of exons. (**C**) Bias of exon length in lincRNAs. Blue and brown violin plots represent the exon length distribution of lincRNA and mRNA containing corresponding number of exons. (**D**) Number of lincRNA on each chromosome. Brown histogram and blue line represent number of lincRNA detected in each chromosome and the length of corresponding chromosome. (**E**) Correlation analysis between chromosome length and lincRNA number in corresponding chromosomes. R^2^ = 0.9142. (**F**) Chromosome distribution of lincRNAs. Chr, chromosome; Mb, Megabase; Gene density represents distribution of mRNAs in each chromosome of Zunla-1_Release_2.0 reference genome; lincRNA density indicating distribution of lincRNAs in each chromosome detected in CK, LDS (*P*. *capsici* inoculation ‘Ledu’), GR (*P*. *capsici* inoculation ‘Ledu’ scion with ‘Jingxin No. 5’ rootstocks) samples, and All samples

Previous studies have shown that both plant and animal lincRNAs are shorter and harbor fewer exons than protein-coding genes [24]. To determine whether pepper lincRNAs share these features, the distribution of length and exon number of 2,388 lincRNAs were analyzed compared with all pepper predicted protein-coding transcripts (35,335 genes from the Zunla-1_Release_2.0 genome, Table S3). Figure 2A shows that 47% of lincRNAs ranged in size from 200 to 1000 nucleotides, with 53% >1000 nucleotides (Table S4). In contrast, for the protein-coding transcripts, 37% were > 1000 nucleotides. Interestingly, most (88%) of the pepper lincRNAs only contained two or three exons, while the number of exons for the protein-coding genes mainly ranged from two to eight (Fig. 2B). For the exon length, most exons in protein-coding genes were longer than exons in lincRNA, but exons in these lincRNA containing two exons were longer than exons in protein-coding genes (Fig. 2C). These results indicated that, unlike protein-coding genes, most of the pepper lincRNAs were relatively longer and contained fewer exons. Regarding chromosome distribution, most lincRNAs were found on Chr00, followed by Chr03 and Chr01 (Fig. 2D). Further analysis revealed that lincRNA numbers were significantly correlated with chromosome length (Fig. 2E, R² = 0.9142 ***). The circos plot clearly showed that pepper lincRNAs were not evenly distributed across chromosomes (Fig. 2F). Similar to protein-coding genes, lincRNAs had higher densities in the euchromatin regions than in the pericentromeric heterochromatin (Fig. 2F). These results suggest that pepper lincRNAs may share similar transcription features with the protein-coding genes. In addition, some lincRNAs were transcribed from loci much closer to the telomeres than protein-coding genes. For instance, some lincRNAs were generated from the ends of Chr02 and Chr07 (Fig. 2F).

### Expression pattern profiling of lincRNAs

**Fig. 3:**
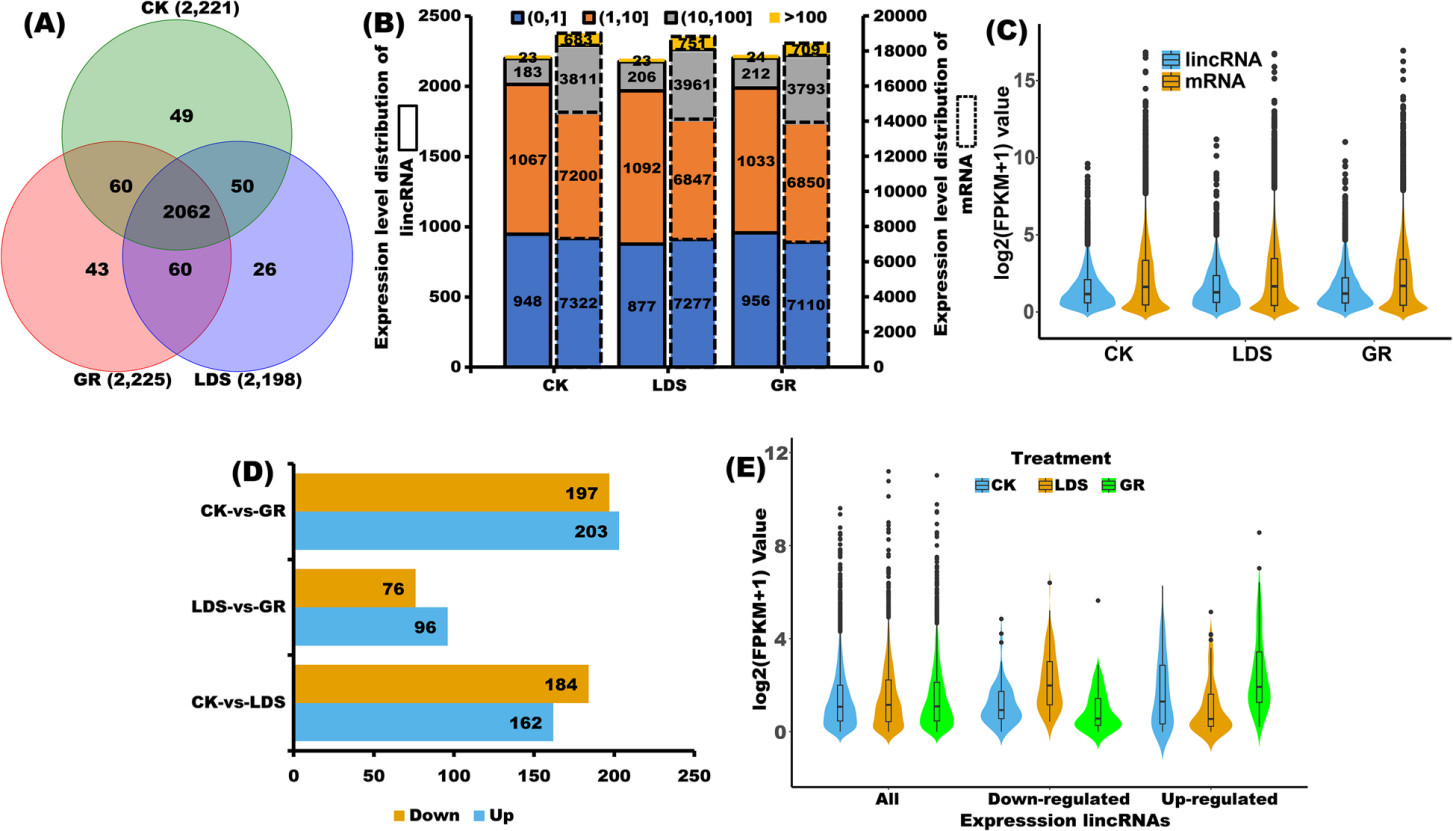
Expression pattern profiling of pepper lincRNAs. (**A**) Venn diagram showing the amount and distribution of lincRNAs in CK (cv ‘Ledu’ inoculation with water), LDS (cv ‘Ledu’ inoculation with *P*. *capsici* spores), and GR (cv ‘Ledu’ scion with ‘Jingxin No. 5’ rootstocks inoculation with *P*. *capsici* spores) samples. (**B**) Expression level distribution of lincRNAs and mRNAs in CK, LDS, and GR samples. (**C**) Expression pattern overview of lincRNAs and mRNAs in CK, LDS, and GR samples. (**D**) Number of down- and up-regulated lincRNAs in pairwise comparisons of CK-vs-GR, LDS-vs-GR, and CK-vs-LDS. (**E**) Overview dynamic change of all expression lincRNA and GR-vs-LDS DE-lincRNAs in CK, LDS, and GR samples

A similar number of lincRNAs were detected in CK (2221, cv ‘Ledu’ inoculation with water), LDS (2198, cv ‘Ledu’ inoculation with *P*. *capsici* spores), and GR (2225, cv ‘Ledu’ scion with ‘Jingxin No. 5’ rootstocks inoculation with *P*. *capsici* spores) samples (Fig. 3A). The expression of lincRNAs was divided into four classes (Fig. 3A): (1) low (FPKM ≤ 1); (2) moderate (1 < FPKM ≤ 10); (3) high (10 < FPKM ≤ 100); and (4) very high (100 < FPKM). In each treatment, the majority of lincRNAs belong to the low (41%) and moderate (48%) classes based on lincRNA expression; however, some lincRNAs belonged to the high or very high classes, indicating that the lincRNAs exhibit a biological purpose, rather than simply representing transcriptional ‘noise’ (Fig. 3B). [35] Based on the expression pattern overview and expression level distribution of lincRNAs and mRNAs, we inferred that (1) lincRNAs commonly display lower expression levels than mRNAs in each sample (Fig. 3C), and (2) the ratio of low expression lincRNAs and mRNAs is similar, whereas the ratio of moderate expression lincRNAs is more than mRNAs, but the ratio of high or very high expression lincRNAs is less than mRNAs (Fig. 3B, C). To systematically explore the transcriptomic dynamics, pair-wised comparisons between different treatments of interest were conducted (Fig. 3D) and differentially expressed lincRNAs (DE-lincRNAs) were identified. In total, 607 DE-lincRNAs were identified in different pairs of treatments, of which 346, 172 and 400 were found between LDS and CK, LDS and GR, and GR and CK, respectively (Fig. 3D). Subsequently, this study focused on those GR-vs-LDS DE-lincRNAs that were possibly responsible for the improving resistance level of grafted peppers and compared their dynamic regulation patterns among GR, LDS, and CK. Interestingly, after grafting cv. ‘Ledu’ scion to cv. ‘Jingxin No. 5’ rootstocks, the expression levels of most of the DE-lincRNAs induced by *P*. *capsici* infection showed the tendency of recovering to the control levels (Fig. 3E).

### lincRNAs mediating the expression of neighboring genes by cis-regulation

**Fig. 4:**
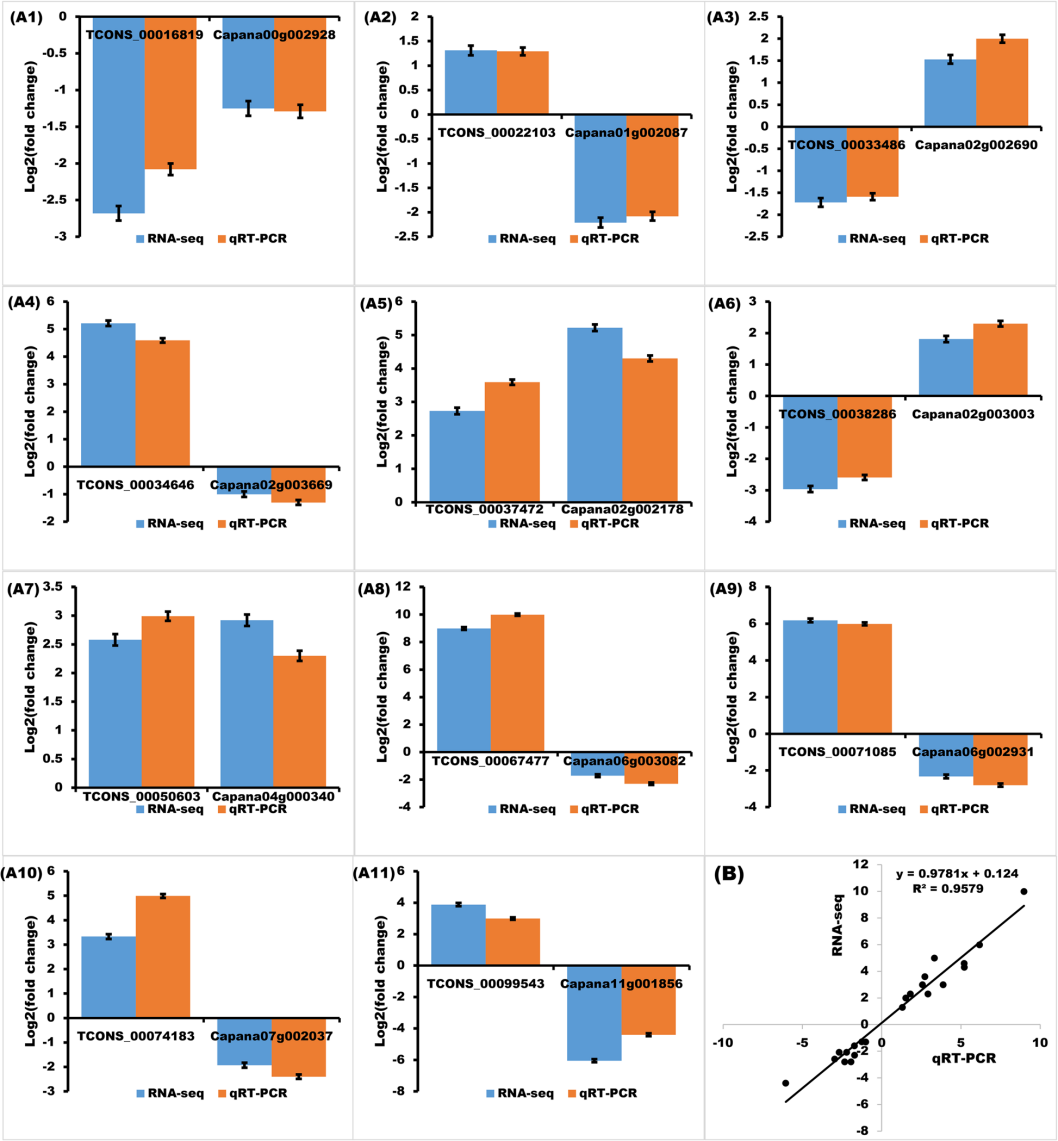
Comparison of regulation patterns obtained from RNA-seq and RT-qPCR data of 11 DE-lincRNAs and 11 potentially *cis*-regulating DE-genes between GR and LDS samples. (**A**) Up- and down-regulation patterns of lincRNAs and mRNAs determined by RNA-seq (blue histogram, details showed in Table 1) and qRT-PCR (red histogram). For RNA-seq, FPKM values were used to calculated the expression level fold change of corresponding lincRNA and mRNA between GR and LDS samples [FPKM(GR)/FPKM(LDS)], then to calculated the log2(fold change) values; for qRT-PCR, the fold-change of corresponding lincRNA and mRNA between GR and LDS samples was calculated as (relative expression under GR treatment)/(relative expression under LDS treatment), then to calculated the log2(fold change) values. (**B**) Correlation analysis of the RNA-seq and qRT-PCR data. The log2(fold change) data determined by RNA-seq (blue) and qRT-PCR (red) in A1-A11 were used to calculate the regression equation and draw the regression curve. R^2^ = 0.9579

Previous studies showed that lincRNAs could play a *cis*-regulatory role by mediating the expression of neighboring genes [36]. Thus, the 10 kb upstream or downstream of the 173 differentially expressed lincRNAs was investigated, and 40 protein-coding genes were close to 40 differentially expressed lincRNAs between GR and LDS samples (Table S5). Among them, 11 protein-coding genes were differentially expressed between GR and LDS samples (Table 1). Then, the expression patterns of DE-lincRNAs and DE-neighboring mRNAs were further confirmed by qRT-PCR. The up- and down-regulation patterns of lincRNAs and mRNAs determined by RNA-seq were consistent with qRT-PCR (Fig. 4. R^2^ = 0.9579 ***), suggesting that: (1) expression patterns of lincRNAs determined by RNA-seq were reliable, and (2) the possibility of DE-lincRNAs *cis*-regulating DE-neighboring genes were further confirmed [27].

**Table 1:** DE-lincRNAs and their corresponding *cis*-regulating DE-neighboring genes

lincRNA_ID	log2(FC)	Gene_ID	Stream	log2(FC)	Description
TCONS_00016819	-2.68	Capana00g002928	Down	-1.25	Phosphatidylinositol-4-phosphate 5-kinase
TCONS_00022103	1.31	Capana01g002087	Up	-2.21	Bnacnng15960d
TCONS_00033486	-1.72	Capana02g002690	Up	1.53	Maf-like protein
TCONS_00034646	5.21	Capana02g003669	Down	-1.00	Pollen allergen Che a 1
TCONS_00037472	2.73	Capana02g002178	Down	5.22	Zinc finger MYND domain-containing protein 15
TCONS_00038286	-2.96	Capana02g003003	Down	1.81	Esterase KAI2-like
TCONS_00050603	2.58	Capana04g000340	Up	2.92	Zinc-finger homeodomain protein 5
TCONS_00067477	8.98	Capana06g003082	Down	-1.71	Pentatricopeptide repeat-containing protein
TCONS_00071085	6.18	Capana06g002931	Down	-2.33	LRR receptor-like serine/threonine-protein kinase
TCONS_00074183	3.33	Capana07g002037	Down	-1.93	Persulfide dioxygenase ETHE1 homolog
TCONS_00099543	3.88	Capana11g001856	Down	-6.05	Maintenance of telomere capping protein 1-like

To further reveal the function of lincRNAs in mediating grated pepper resistance to *P*. *capsici*, function annotation to DE-neighboring genes was performed. As shown in Table 1, in this study, we found Capana00g002928, a down-regulated phosphatidylinositol-4-phosphate 5-kinase, could be *cis*-regulated by down-regulated lincRNA TCONS_00016819. Phosphoinositide phosphate kinases are implicated in membrane trafficking and are important for plant growth, development, and the immune responses [37]. For example, phosphatidylinositol-4 kinases PI4Kβ1/β2 plays important roles during salicylic acid-mediated plant defense signaling in Arabidopsis [38]. Zinc finger-coding genes play important roles in the regulation of growth and development, hormone signaling, and responses to biotic and abiotic stress in plants. For example, down-regulation of tomato *Sly-TG2* can increase the susceptibility of leaves to *P*. *infestans* infection [39]. Consistently, in this study, zinc finger proteins Capana02g002178 and Capana04g000340, which were potentially *cis*-regulated by lincRNA TCONS_00037472 and TCONS_00050603, were up-regulated by *P*. *capsici* infection in resistant grated pepper plants.

Pentatricopeptide repeat-containing proteins (PPRs) contribute profound effects on organelle biogenesis and function and, consequently, on photosynthesis, respiration, plant development, and environmental responses [40]. In Arabidopsis, six PPRs (PPR40, ABO5, AHG11, SLG1, PGN, and SLO2) have been reported to regulate ABA signaling and salt or drought stress responses [41]. Two PRRs, PPR1 and PPR2, were up-regulated upon pathogen challenge, and their knockdown mutants displayed much more severe disease symptoms than wild-type plants [42]. Consistently, in this study, a pentatricopeptide repeat-containing protein coding gene *Capana06g003082* which were potentially *cis*-regulated by lincRNA TCONS_00067477, was differently regulated by *P*. *capsici* infection in resistant grated pepper plants.

LRR receptor-like serine/threonine-protein kinases (RLPs) play fundamental roles in plant defense signaling cascades [43]. For example, several cloned leaf mold resistance genes of tomato, Cf-2, Cf-4, Cf-5, and Cf-9, are typical RLPs [44]. In this study, Capana06g002931, an LRR receptor-like serine/threonine-protein kinase that was potentially *cis*-regulated by lincRNA TCONS_00071085, was down-regulated by *P*. *capsici* infection in resistant grated pepper plants.

### Predicted lincRNA-miRNA-mRNA interaction networks

**Fig. 5:**
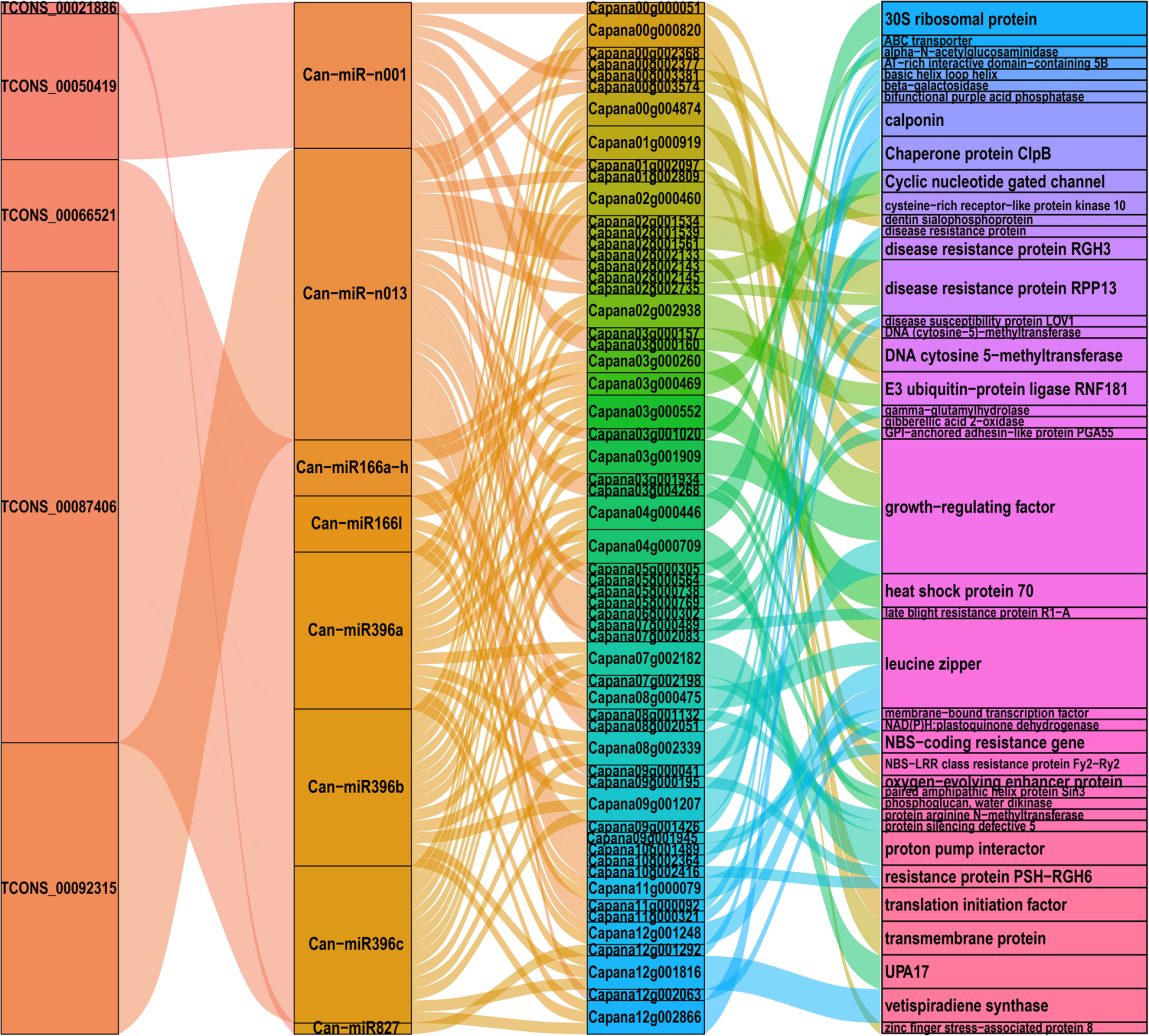
Sankey diagram for the lincRNA-miRNA-mRNA interaction networks. The four columns represent lincRNA, miRNA, mRNA, and mRNA function annotation. Each rectangle represents one gene, and the connection degree of each gene is illustrated according to the size of the corresponding rectangle

LincRNAs also function as endogenous target mimics (eTMs) and can competitively bind some specific miRNAs, which provide a new mechanism for lincRNAs-regulated mRNA expression through lincRNA-miRNA-mRNA interaction networks [5, 45]. To further uncover the functions of lincRNAs in mediating grafted pepper resistance to *P*. *capsici*, the miRNAs bound by lincRNAs, and mRNAs targeted by miRNAs were predicted. Among 171 differentially expressed lincRNAs, five could potentially bind nine miRNAs, and these miRNAs targeted 59 differentially expressed mRNAs (Fig. 5, Table S6, S7). Previously, Kwenda, et al. [5] found that potato lincRNAs were potentially targeted by several miRNAs that were implicated in plant immune defences. Consistently, in this study, based on the function annotations of mRNAs, some genes that were reported to play important roles in plant resistance to pathogens were potentially regulated by lincRNAs through lincRNA-miRNA-mRNA interaction networks. For example, lincRNA TCONS_00087406 was found to act as an eTM and could potentially bind to pepper miRNA can-miR396a, can-miR396b, and can-miR396c, thus further regulating the expression of growth regulating factors *Capana01g000919*, *Capana02g002938*, *Capana03g001909*, and *Capana08g002339*. It was previously reported that MiR396 is a highly conserved microRNA (miRNA) family targeting growth regulating factor (GRF) genes [46]. In rice, miR396 negatively regulates rice blast disease resistance by suppressing multiple *OsGRFs*, which in turn differentially control growth and yield [46]. In this study, TCONS_00087406 was up-regulated in resistant grafted pepper, and *Capana01g000919* and *Capana08g002339* were also up-regulated, which suggested that TCONS_00087406 competitively bound miRNAs to release the suppression of mRNA expression. E3 ubiquitin-protein ligases, also known as RING finger proteins, function as ubiquitin ligases and play key roles in biotic and abiotic stress. For example, *StRFP1* contributes to broad-spectrum resistance against *P. infestans* in potato [47]. In this study, TCONS_00050419 was found to potentially bind to can-miR-n001, which could target the E3 ubiquitin-protein ligases Capana00g000051, Capana03g000157, and Capana03g000160. Considering the expression patterns, TCONS_00050419 and three E3 ligases were all up-regulated in resistant grafted pepper, implying that lincRNAs competitively binds miRNAs to release the suppression of mRNA expression. It was reported that phytohormone balance plays a central role in the outcome of plant-pathogen interactions. For example, pepper *CaGA2ox1* plays a role in plant defense signaling and plant-microbe interactions, thus increasing the host resistance response [48]. In this study, a gibberellic acid 2-oxidase coding gene *Capana01g002809* was found to be targeted by can-miR-n013, and the miRNA was competitively bound by lincRNA TCONS_0009231, indicating the role of lincRNAs to enhanced grafted pepper resistance by mediating the expression of phytohormone-associated genes. Interestingly, lincRNA TCONS_00092315 bound to can-miR-n013, and this miRNA targeted a disease susceptibility protein LOV1 Capana11g000321. It has been reported that, as a member of the NBS-LRR resistance gene family, LOV1 contributes to disease susceptibility in Arabidopsis [49]. In this study, consistent with the phenotype of grafted pepper resistance to *P*. *capsici*, *Capana11g000321* was down-regulated in grafted plants. Furthermore, lincRNA TCONS_00092315 was also down-regulated, suggesting a regulating network in which TCONS_00092315 releases miRNA can-miR-n013 to suppress the expression of susceptibility gene Capana11g000321, thus increasing the resistance in grafted pepper to *P*. *capsici*.

[50]

## Conclusions

In this study, genome-wide identification and characterization of pepper lincRNAs were performed. In total, 2,388 reliable lincRNAs were detected. They were relatively longer and contained fewer exons than protein-coding genes. Similar to coding genes, lincRNAs had higher densities in the euchromatin regions and longer chromosome transcribed more lincRNAs. Expression pattern analysis suggested that lincRNAs commonly display lower expression levels than mRNAs. Totally, 607 differentially expressed lincRNAs (DE-lincRANs) were identified, of which 172 were found between *P*. *capsici* resistant grafted pepper sample GR and susceptible sample LDS. The neighboring genes of DE-lincRNAs and miRNAs competitively sponged by DE-lincRNAs were identified. Function annotation revealed that neighboring genes, such as phosphoinositide phosphate kinases, zinc finger proteins, pentatricopeptide repeat-containing proteins, and LRR receptor-like serine/threonine-protein kinases, are *cis*-regulated by DE-lincRNAs. In addition, genes coding growth regulating factors, E3 ubiquitin-protein ligases, gibberellic acid 2-oxidases, disease susceptibility protein LOV1, among others, were regulated by DE-lincRNAs through lincRNA-miRNA-mRNA interaction networks. Overall, the study identified pepper lincRNAs and suggested their potential roles in increasing the resistance level of grafted pepper to *P*. *capsici*.

## Supplementary Information

**Additional file 1: Table S1.** Detailed information on primers for qRT-PCR. **Table S2.** Expression profiles of lincRNAs represented by FPKM. **Table S3.** The gff annotation of lincRNAs. **Table S4.** Sequences of lincRNAs. **Table S5.** Differentially expressed lincRNA and their up/down stream mRNAs. **Table S6.** Differentially expressed lincRNA and their binding miRNAs. **Table S7.** The miRNAs and their targeting mRNAs.

## Data Availability

RNA-seq reads were deposited in NCBI SRA database under project accession PRJNA728756. All data generated or analyzed during this study are included in this published article and its Additional files. The datasets generated and analyzed during the current study are available from the corresponding author on reasonable request.

## Abbreviations

ncRNAs: noncoding RNAs
lncRNAs: long ncRNAs
lncNATs: natural antisense long noncoding RNAs
lincRNAs: long intergenic ncRNAs
DE-lincRANs: differentially expressed lincRNAs
GR: *P*. *capsici* inoculation ‘Ledu’ scion with ‘Jingxin No. 5’ rootstocks
LDS: *P*. *capsici* inoculation ‘Ledu’
CK: control of non-inoculated ‘Ledu’
FPKM: fragments per kilobase of exon per million fragments mapped
PCA: principal component analysis
qRT-PCR: quantitative real-time PCR
